# Esophageal Melanocytosis

**DOI:** 10.4322/acr.2024.487

**Published:** 2024-05-22

**Authors:** Samikshya Thapa, Gajendra Kumar Yadav, Ratna Mondal, Ravi Hari Phulware

**Affiliations:** 1 All India Institute of Medical Sciences, Department of Pathology & Laboratory Medicine, Rishikesh, Uttarakhand, India

**Keywords:** Esophagus, Endoscopy, Gastrointestinal, Esophageal Mucosa, Melanins, Gastroesophageal Reflux

## Abstract

Esophageal melanocytosis is a rare entity defined by the proliferation of a melanocytic basal layer of the esophageal squamous lining and deposition of melanin in the esophageal mucosa. Esophageal melanocytosis is considered a benign entity of unknown etiology; however, it has been reported as a melanoma precursor. We report a case of esophageal melanocytosis in a diabetic and hypertensive 67-year-old male with recurrent dizziness and syncope for the past 6 months. Given his complaint of dyspepsia, he underwent an upper gastrointestinal endoscopy, in which an esophageal biopsy revealed the diagnosis of esophageal melanocytosis. The definitive diagnosis of esophageal melanocytosis can only be made by histological analysis. The histologic differential diagnoses include melanocytic nevi and malignant melanoma. Therefore, they need to be ruled out.

## INTRODUCTION

Esophageal melanocytosis is a rare entity characterized by the proliferation of the melanocytic basal layer of the esophageal squamous lining and the deposition of melanin in the esophageal mucosa.^1^ Typically, these lesions are detected by endoscopic screening, and only 0.07-2.1% are observed in gastrointestinal endoscopies.^2^Typically, esophageal mucosa is devoid of melanocytes. However, abnormal migration of melanocytes to the esophageal mucosa during the embryonic life or keratinocyte differentiation of the esophageal multipotential stem cells in the basal epithelial layer is considered.^3,4^ While typically asymptomatic, it can raise concerns due to lack of diagnosis and its potential association with premalignant and malignant lesions.^3^

Due to its rarity and similarities to other mimickers, it cannot be distinguished based on endoscopic grounds, requiring histopathologic with immunohistochemistry (IHC) studies. Herein, we present a case of esophageal melanocytosis in an adult patient with dyspeptic symptoms who had been experiencing recurrent dizziness and syncope for six months in the setting of hypertension and diabetic mellitus.

## CASE REPORT

An apparently healthy 67-year-old male presented with a history of recurrent dizziness and syncope for 6 months, which became more frequent over the last 3 months. He was on regular medication for hypertension and diabetes mellitus, diagnosed 1 year back. He was a non-alcoholic, non-smoker, and had no history of weight change or previous surgery.

His physical examination was unremarkable. His vitals were within normal limits. The laboratory workup revealed a normal hemogram, random glucose, glycated hemoglobin, serum electrolytes, and liver and renal function tests.

**Figure 1:**
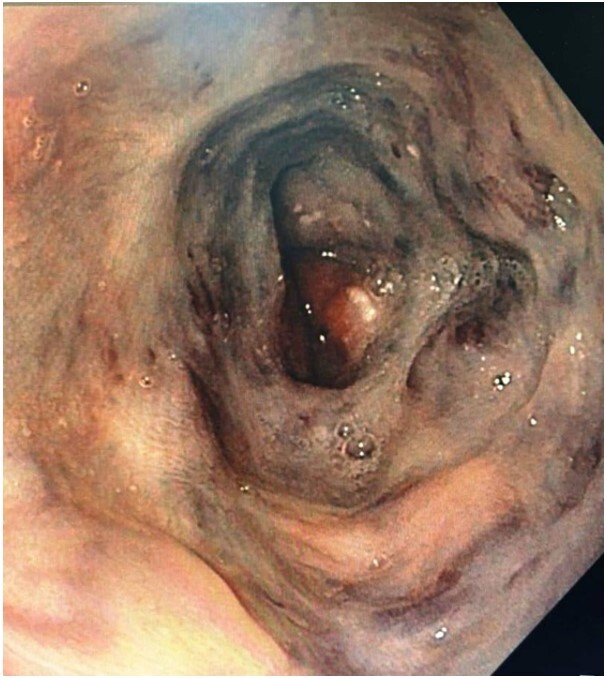
Photomicrograph of the upper gastrointestinal tract endoscopy shows darkly pigmented, flat irregular lesions.

Chest X-ray was unremarkable. His electrocardiogram showed intermittent complete heart block, which required a permanent pacemaker implantation. He was subjected to upper gastrointestinal tract endoscopy as a part of evaluation for dyspepsia, which revealed black macular spots that involved the whole esophageal mucosa (Figure 1) extending from the proximal to the distal esophagus and multiple sessile gastric polyps, mainly in the fundus and body.

**Figure 2:**
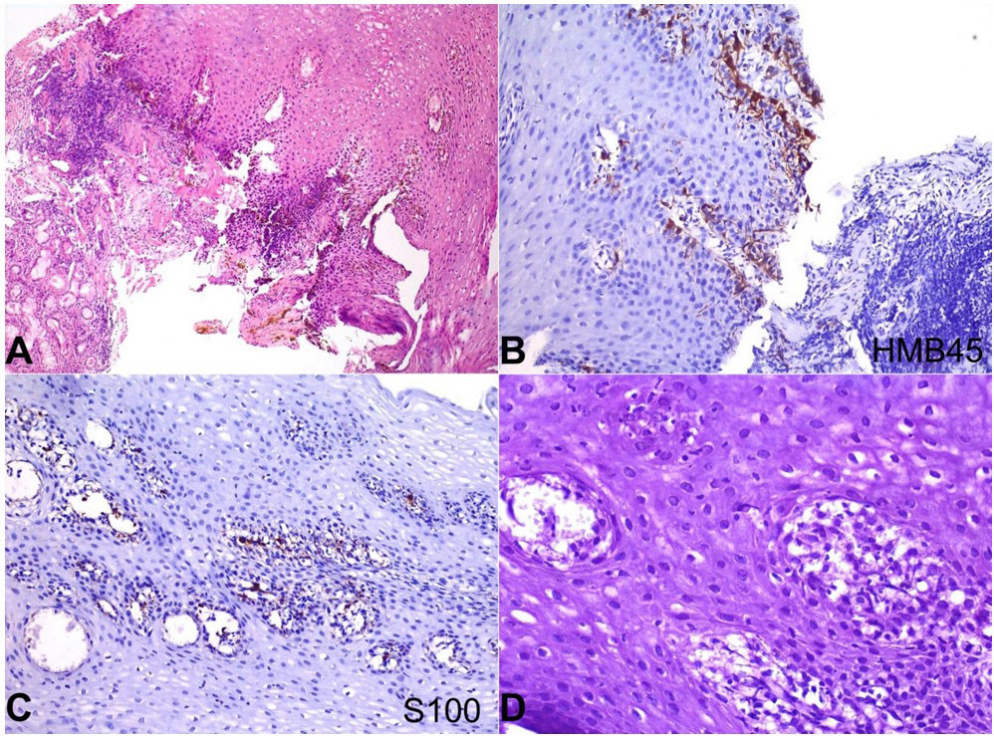
Photomicrograph from gastroesophageal junction biopsy shows in **A –** Gastric mucosal glands and hyperplastic stratified squamous epithelium. The basal layer of squamous epithelium shows an increased number of pigment-laden dendritic melanocytes and an increased quantity of melanin pigment (H&E x200); **B** and **C –** These intraepithelial melanocytes are immunopositive for HMB45 and S100, respectively (B x400; C x200); **D –** Special stain for melanin bleach using hydrogen peroxide shows completely bleached melanin pigment.

Multiple biopsies were taken from the esophageal pigmented areas and the gastric mucosa. Microscopic examinations showed esophageal tissue lined by hyperplastic stratified squamous epithelium with an increased number of pigment-laden melanocytes and deposition of coarse brownish-black pigment within the basal layer. These melanocytes showed no cellular and nuclear atypia. The underlying lamina propria showed similar melanocytes and chronic inflammatory infiltrate (Figure 2A). No dysplasia or features of GERD were noted. On immunohistochemistry, these cells were immune-reactive for HMB45 (Figure 2B) and S100 (Figure 2C). The melanocytes were bleached entirely with hydrogen peroxide, suggesting the pigment was melanin (Figure 2D). Also, these cells were negative for the periodic acid Schiff (PAS) and Perl’s iron stain. The polyps were histologically diagnosed as hyperplastic, and the remaining gastric mucosa demonstrated normal architecture. No evidence of melanomas or other malignancies was found.

The final diagnosis was released as esophageal melanocytosis based on histomorphological features and special staining combined with endoscopic findings.

The postoperative period following cardiac pacemaker implantation was uneventful, and the patient presented a favorable outcome in the follow-up.

## DISCUSSION

Esophageal melanocytosis (EM) is a rare entity characterized by the deposition of melanin pigment in the esophageal mucosa and the proliferation of melanocytes in the basal layer. It was first described by De la Pava et al.^5^ in 1963 as scattered melanocytes at the interface between the epithelium and the lamina propria of the esophageal mucosa. The overall prevalence of EM was reported to be 4% in autopsy cases, while 0.1% in Japan and 2.1% in India in endoscopic screenings.^6^

Although most earlier papers used the term "melanosis," this term should be avoided as it does not adequately characterize the increased number of melanocytes or specify the melanin nature of the pigment.^7^

EM is more frequent in adults, with a slight male predominance. It is often an incidental finding during routine endoscopic examinations that mainly addresses the esophagus’s middle and lower portions.^8^ Melanocytes originate in the neural crest during embryonic development and migrate through the peripheral nerves to the skin and squamous mucosa during fetal life. Typically, the esophagus has no melanocytes.^4^ The etiology and pathogenesis of EM remain uncertain. However, several theories have been proposed, including the" Esophageal melanocytosis may be a result of entrapment of melanin from refluxed gastric content, gastroesophageal reflux disease, and other chronic stimuli which cause mucosal damage and keratinocytes hyperplasia".^2^ While other theories include the Melanocyte migration from adjacent structures, Aberrant migration of the melanocytes during embryogenesis, Melanin production by epithelial cells because of differentiation from the stem cells in the basal layer of the squamous epithelium. ^9,10^

In a study of esophageal squamous cell carcinoma in situ, Ishida et al.^11^ discovered that the melanocytosis pigment was present in non-neoplastic squamous epithelium in 3.8% of patients. Melanocytosis of the basal epithelial layer may contribute to the formation of primary malignant melanoma, depending on the severity of chronic esophagitis. According to Walter et al.^12^melanocytosis has highlighted the need to be taken into consideration in the development of esophageal malignancy. Additionally, Yokoyama et al.^3^ revealed that esophageal dysplasia, esophageal melanoma, and esophageal cancer are preceded by esophageal melanosis.^3^ The Maroy et al.^13^ study described the occurrence of melanoma in a patient who had been followed up on for eight years and had been diagnosed with benign melanocytosis in the esophagus.

Esophageal melanocytosis is frequently symptomless. However, when symptomatic, it may elicit odynophagia, heartburn, and dysphagia. It might occasionally be linked to esophageal strictures or gastrointestinal hemorrhage.^11^The diagnosis of EM is primarily based on endoscopic findings. Endoscopically, EM has been described as flat, oval, and irregularly delineated blackish patchy pigmented lesions. Electron microscopy shows the presence of dendritic melanocytes with melanosomes in various stages of development in the basal layer of the esophageal epithelium.^8^ A biopsy is necessary to confirm the diagnosis and rule out other disorders, including melanoma and melanosis coli. For esophageal melanocytosis, there is no particular therapy. The goal of management is to treat any underlying medical issues and any related symptoms. Endoscopic ablative procedures may be utilized occasionally to treat bleeding or esophageal strictures.^15^

The differential diagnoses for esophageal melanocytosis include (i) Primary malignant melanoma of the esophagus, accounting for 0.1-0.4% of all esophageal neoplasms. Endoscopically, melanoma presents as a pigmented or non–pigmented polypoidal mass in the middle and lower esophagus.^12^ The biopsy helps differentiate EM from melanoma, as the EM exhibits benign melanocytic cells; (ii**)** Benign melanocytic nevi are highly uncommon in esophageal mucosa. To date, only a single case of blue nevus was reported by Lam et al.^13^ in a 52-year-old Chinese woman who presented with linear patches of bluish pigmentation in the lower esophagus;^13^ (iii) Black esophagus is a rare observation in upper endoscopy with ulceration corresponding to severe acute inflammation with mucosal necrosis seen on histologic examination. It is generally associated with ischemic injury in diabetic patients;^14^ (iv) Anthracosis - Esophageal pigmentation can result from coal dust exposure, particularly in a history of previous occupational exposure;^15^ (v) Drug-induced pigmentation - Certain medications, like minocycline, can cause mucosal pigmentation, requiring a comprehensive medication assessment.^16^ Exogenous dye ingestion, hemosiderosis, and pseudomelanosis are the other differentials.^14-15^

“Smoker melanosis” has been reported to occur in approximately 20% of the oral mucosa of smokers in the Western and Asian populations and the mucosa of the upper aerodigestive tract, the oropharynx, laryngopharynx, and esophagus. This results from prolonged damage to the mucosa of the upper gastrointestinal tract, especially concerned with reverse smoking.^17^

## CONCLUSION

This case report highlights the importance of considering esophageal melanocytosis in the differential diagnosis of dyspepsia, particularly in elderly patients with esophageal symptoms. Despite its benign nature, further research is thus vital to unveil the mechanisms behind this unique pigmentation and clarify its clinical impact. Recognition of esophageal melanocytosis is crucial to avoid unnecessary interventions and ensure appropriate management strategies for affected individuals. Long-term follow-up investigations are necessary to track the development and possible consequences of this unusual esophageal condition, including malignant transformation.
